# Computer-Aided Approach for Rapid Post-Event Visual Evaluation of a Building Façade

**DOI:** 10.3390/s18093017

**Published:** 2018-09-09

**Authors:** Jongseong Choi, Chul Min Yeum, Shirley J. Dyke, Mohammad R. Jahanshahi

**Affiliations:** 1 School of Mechanical Engineering, Purdue University, West Lafayette, IN 47907, USA; choi343@purdue.edu (J.C.); sdyke@purdue.edu (S.J.D.); 2 Department of Civil and Environmental Engineering, University of Waterloo, Waterloo, ON N2L 3G1, Canada; 3 Lyles School of Civil Engineering, Purdue University, West Lafayette, IN 47907, USA; jahansha@purdue.edu

**Keywords:** post-event visual evaluation, image localization, orthophoto generation, unmanned aerial vehicle

## Abstract

After a disaster strikes an urban area, damage to the façades of a building may produce dangerous falling hazards that jeopardize pedestrians and vehicles. Thus, building façades must be rapidly inspected to prevent potential loss of life and property damage. Harnessing the capacity to use new vision sensors and associated sensing platforms, such as unmanned aerial vehicles (UAVs) would expedite this process and alleviate spatial and temporal limitations typically associated with human-based inspection in high-rise buildings. In this paper, we have developed an approach to perform rapid and accurate visual inspection of building façades using images collected from UAVs. An orthophoto corresponding to any reasonably flat region on the building (e.g., a façade or building side) is automatically constructed using a structure-from-motion (SfM) technique, followed by image stitching and blending. Based on the geometric relationship between the collected images and the constructed orthophoto, high-resolution region-of-interest are automatically extracted from the collected images, enabling efficient visual inspection. We successfully demonstrate the capabilities of the technique using an abandoned building of which a façade has damaged building components (e.g., window panes or external drainage pipes).

## 1. Introduction

During tornado or hurricane events, nonstructural components on the façades or roofs of buildings are highly vulnerable to strong winds or airborne debris. These often cause serious damage to the building components, leading to disruption in the functions of the building and, potentially, jeopardizing the safety of its occupants. Moreover, damage on cladding (e.g., spalling or crack), their anchorage to the walls, or window panes (e.g., crack or dislocation) may induce dangerous falling hazards to pedestrians on the sidewalk below, followed by restrictions on the use of the adjacent roads. Thus, inspection of building façades is one very important task conducted during disaster recovery and must be conducted in a rapid manner [1].

Regardless of the degree of urgency, manual visual observation by human engineers is still the primary method for façade inspection [2]. Currently, several human inspectors are needed to physically visit every floor of each building. Then, those inspectors evaluate the condition (e.g., crack or dislocation) of each component on the façade and annotate them on the corresponding engineering drawing (or layout), producing a damage map for each façade. In the worst cases, special equipment may be required to access the building from the outside to inspect the condition of the building’s exterior, such as ladders or ropes for controlled decent. Such manual process would become extremely tedious and inefficient at the site. However, unfortunately, there is not a viable rapid technique to streamline this important process.

Recent advances in image sensors and sensing platforms have been achieved, enabling automated or semi-automated vision-based visual inspection [3,4,5,6,7,8,9,10,11]. Incorporating vision sensors onto aerial sensing platforms will alleviate the spatial and temporal limitations that are typically associated with manual inspection in the case of large-scale buildings [12,13,14,15,16,17,18,19]. For example, unmanned aerial vehicles (UAVs) can collect a large volume of high-resolution images to cover the entire area of buildings in an efficient manner. With automated methods to use and process this data, computer-aided inspection can overcome time-consuming and risky human-based inspection for the building façades.

However, a major technical challenge in using those images for façade inspection is considering the trade-offs between the need for collecting close-up images and their localization. If the images are collected at a close distance to the building façades, it is difficult to know where the corresponding images were captured from. Manual searching is laborious when the building façades are large or have repetitive patterns. Although GPS can be measured and recorded on the images, it does not provide sufficient accuracy regarding the camera pose and location (a typical error range is 5–15 m), and the signal is often interrupted by the roof and/or wall of the buildings [20]. On the other hand, if the images are taken far from the building facade, vision-based inspection may not be feasible due to a lack of details. For instance, to capture crack damage, it is necessary to collect images at a close distance and under different viewpoints and positions for accurate inspection. These requirements are because the visibility of the crack depends on the viewing angles. Since UAVs do not selectively capture favorable images in an automated manner, a large volume of images needs to be collected and used for visual inspection [19,21,22,23]. Thus, to enable efficient visual inspection using such a large volume of images, an automated technique should be incorporated to localize the close-view images captured from different viewpoints to the corresponding region on the building facades.

In this study, we envision a new inspection procedure for the building façade as follows: After an event, inspectors fly UAVs manually or autonomously using GPS from a place that is safe from falling hazards. The UAVs capture a large volume of images of the façades of the building. Once the images are collected, we automatically construct a high-resolution orthophoto using those images. This orthophoto allows the inspectors to readily view the entire building façade. Then, they select any region on the orthophoto that is suspicious or vulnerable to damage. The high-resolution image patches corresponding to the selected region, named regions-of-interest (ROIs), are extracted from the original images, enabling quality visual assessment. With this approach, the inspector can evaluate several buildings in just a short time, saving time that would be spent physically visiting each building. Moreover, it is a much safer way to conduct an inspection of multiple damaged buildings without actually accessing them.

To implement this new procedure, we have developed a technique to support rapid visual inspection of planar building façades using images collected by UAVs. An orthophoto of each building façade is automatically constructed using a structure-from-motion (SfM) technique followed by image stitching and blending. The resulting orthophoto contains an entire view of the building façade so that inspectors can easily recognize and select target regions for inspection (TRIs). This orthophoto is geometrically connected to each of the original images. Thus, once the engineers select the TRIs on the orthophoto, all image regions containing the corresponding TRIs, or the ROIs, are automatically identified and extracted from the original images. This approach will directly support field engineers in conducting rapid inspection. To demonstrate the capabilities of the technique developed, we collect images from a damaged façade of an actual building using UAVs. We successfully generate the high-resolution orthophoto of the façade and extract the ROIs corresponding to the selected TRIs including a damaged window pane and external drainage pipe.

The major advantage of the technique is that it can rapidly provide the orthophoto and localized ROIs from a raw collection of images for robust visual inspection. The orthophoto is useful for making annotation and documentation of damage locations because it provides an entire view of the building façade. This can streamline the current time-consuming process of manual documentation by human inspectors. Also, applying an existing damage detection algorithm to the ROIs may fully automate the inspection process. Such use of highly relevant ROIs greatly reduces false-positive and false-negative damage detection by processing many images captured from various viewpoints for viewing [19,21,22,23].

The remainder of this paper is organized as follows. Section 2 begins with an overview of the technique and introduces the details of the technical steps, regarding image acquisition, orthophoto generation, and ROI localization. In Section 3, a case study is presented to demonstrate the capability of the technique using images collected from a full-scale building which contains damaged components on its facades. Section 4 contains the discussion and conclusions.

## 2. System Overview

**Figure 1:**
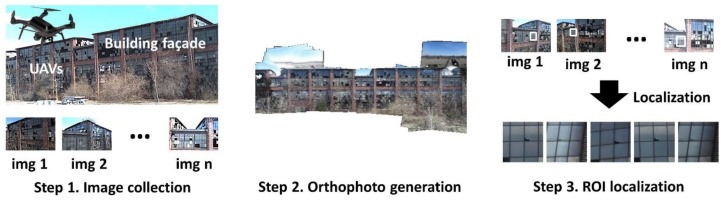
System overview: Step 1. Image collection—collecting many close-up images to cover the entire area of a building façade under various viewpoints, Step 2. Orthophoto generation—constructing an orthophoto of the building façade and computing a geometric relationship between the constructed orthophoto and each image, and Step 3. Regions-of-interest (ROI) localization—extracting the ROIs from the images.

An overview of the technique developed here is presented in Figure 1. The objective of the technique is to aid field engineers to perform rapid visual inspection of the target building façades (TBF) through a sequence of images collected using UAVs. The technique can be used to inspect any target components (e.g., broken window panes, spalling concrete, etc.) located on a relatively flat surface of the TBF.

Step 1 is to fly the UAVs over the TBF to collect a large number of high-resolution images. To increase the likelihood of detecting damage, these images are captured from many different viewpoints and positions [19,21,22,23]. The entire region of the building façade that is needed for inspection should be covered by the images. Step 2 is to generate an orthophoto of the TBF from the collected images. The SfM technique is used to estimate the geometric relationship between each image and the TBF. By extracting and matching the visual features, this process conducts calibration of the camera parameters for each image including a projection matrix and radial distortion coefficient(s) as well as generating a 3D point cloud of the scene [24,25,26]. The surface of the TBF can be automatically detected by fitting a plane to the 3D point cloud. Then, the orthophoto is constructed by projecting each image onto the detected plane, followed by stitching and blending them. Lastly, in Step 3, the ROIs corresponding to the TRIs that the engineers select for inspection are extracted from the original images. The geometric relationship with each of the selected TRIs on the orthophoto and the original images is used to localize the corresponding ROIs. Since the localized ROIs are a set of image patches cropped from those images, they contain detailed visual information of the TRIs. Thus, the extracted ROIs enable robust vision-based visual assessment of the facades. Both the orthophoto generation and ROI extraction are developed to be fully automated without the need for manual manipulation. The only manual step is associated with the TRI selection on the orthophoto in Step 3.

### 2.1. Image Collection

In this technique, collecting high-quality aerial images is crucial for accomplishing successful orthophoto generation and ROI localization. The approach developed does not involve any manual process to choose favorable images among the raw images collected with the UAVs. It also does not require any configuration of the parameters in the middle of the process. Thus, the quality of the original images directly affects the accurate extraction and localization of the ROIs for visual inspection. Here, we suggest some important guidelines for the best use of the technique.

First, the images must cover the entire region of the TBF. Because the images are collected with and stored in UAVs, there is no way for engineers to check if all images thoroughly cover the TBF with sufficient quality. Thus, a well-established flight plan is prepared in advance depending on the shape and size of the TBF so that they can readily collect quality images on site. In general, engineers draw a virtual grid of the flight path on the entire area of the TBF and images are collected at a regular interval by following this grid. A depth (distance between the UAV and the TBF) is determined based on the minimum resolution required in the images for effective visual inspection. The smaller the field of view (coverage) becomes, the higher the spatial resolution of the scenes containing the TBF, although more images would need to be captured to cover the entire TBF. The spacing (interval) of the images collected along the flight path is another important parameter and is directly related to the next guideline.

Second, the image collection interval is carefully designed to ensure there is sufficient overlap between adjacent images. Increasing the number of feature matches across multiple images is crucial for computing accurate geometric relationships between the images and the scene using the SfM technique. As a rule of thumb, more than 60% overlap with the adjacent images is recommended. However, this value varies depending on the image quality (e.g., resolution or signal-to-noise) as well as scene characteristics (e.g., unique texture) [27]. To obtain sufficient and constant overlap, we suggest that images be captured using a regular time interval (e.g., continuous shoot mode in regular cameras) under a constant flight speed.

Third, motion blur should be avoided. Motion blur is common in aerial image collection. It occurs when the object being recorded moves relative to the camera during the period of exposure. Large relative movement can produce a lack of sharpness or artifacts (e.g., ghosting) on the images collected. To avoid this problem, translation, and angular movements of the camera (with respect to the scene) should be minimized while the shutter is open. Multiple factors may affect motion blur including environmental conditions (e.g., low light or high wind), UAV platforms (e.g., fast flying speed or non-isolated platform vibration), and camera parameters (e.g., long focal length or low shutter speed). To prevent taking blurry images, we recommend (1) flying UAVs under good weather conditions (e.g., enough daylight and no wind) and with a slow speed, (2) isolating the vibration of the camera with respect to the UAV platform using a multi-axis gimbal or vibration damper (e.g., rubber pad), (3) decreasing the exposure time without increasing the camera’s ISO because a higher ISO produces higher light sensitivity but also more noise, and (4) zooming out the camera and maintaining a short focal length so that the relative scene change due to sudden angular vibration is minimized.

Finally, from the visual inspection standpoint, images should be collected from a variety of viewpoints [21]. Facilitating the observation of the TRI from various angles through the ROIs is a key benefit of the technique developed. To collect images that contain many viewpoints, engineers should fly UAVs following the designed flight path multiple times while using different camera angles each time. Alternatively, one can use a programmable gimbal so that the angle of the camera is cyclically changed during one flight. Such angled images are also valuable because they serve to improve the performance of the SfM technique [28]. Since angled images contain more of the background scene, they provide more overlap with the other images, producing more accurate parameter estimation using the SfM technique, as mentioned in the second point above.

### 2.2. Orthophoto Generation

An orthophoto is a planar image created by arranging and stitching the set of collected images after removing perspective and radial distortions [29]. Since the resulting orthophoto has a uniform scale in each direction, it will show a true aspect ratio of the target regions on the plane (a single façade surface, in this case). Herein, we describe the process needed to construct a high-quality orthophoto from the aerial images collected and to identify the geometric relationship between each image and the orthophoto. With the orthophoto available, engineers can readily view specific areas of the TBF to select the TRIs for visual inspection.

**Figure 2:**
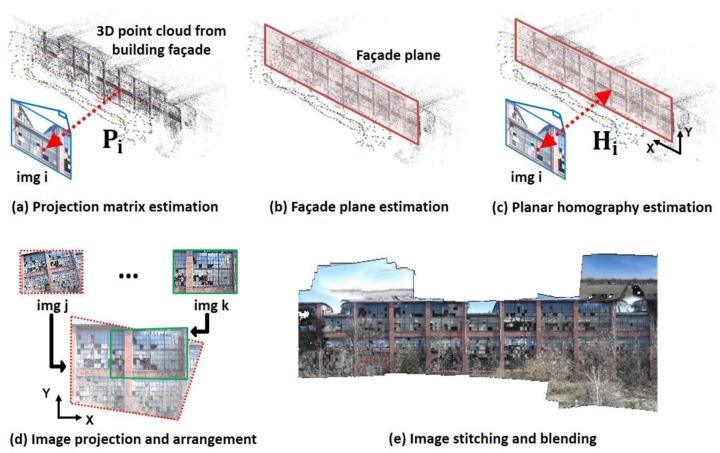
Overview of the orthophoto generation: (**a**) Projection matrix estimation for each image using structure-from-motion (SfM) technique, (**b**) Façade plane estimation from a 3D point cloud, (**c**) Planar homography estimation from corresponding projection matrix, (**d**) Image projection and arrangement into the estimated façade plane (parallel to the XY-plane), and (**e**) Image stitching and blending to generate the complete orthophoto.

First, a projection matrix, **P**, is computed for each image collected using the SfM technique [24]. SfM automatically computes the 3D point cloud and the geometric relationship between the 2D image points and the 3D points in the world (scene). The SfM process first extracts features from each collected image and matches common features among the images. Then, based on the matched features among the images, the relative positions and angles of each image are estimated, and represented as a simple projection matrix. All these results are generated solely from the set of images collected and no manual configuration is required. Only good quality images having enough overlap with the other images are automatically selected and utilized in the SfM process. The SfM process is shown in Figure 2a, where the geometric relationship is represented with the projection matrix, denoted as $P_{i}$ and the subscript indicates an image index. With this matrix, any 3D point in the world can be mapped to its corresponding 2D point in each image. This relationship is represented as

(1) $$x_{i}=P_{i}X$$

where X is any 3D point in the world, and $x_{i}$ is the corresponding 2D image point in image *i*. These equations are established in homogenous coordinates. Thus, X and $x_{i}$ are 4 × 1 and 3 × 1 vectors, respectively. $P_{i}$ is a 3 × 4 matrix that includes information about the camera location and orientation in 3D coordinates (extrinsic orientation) and internal camera parameters (camera calibration matrix), describing the mapping of 3D points in the world to 2D points in each image. Note that the relationship in Equation (1) is valid under the assumption of a pin-hole camera model [24]. Thus, lens distortion of the images should be corrected in advance. Engineers can use a calibrated camera or can correct their distortion using lens distortion parameter(s) computed from SfM.

Second, the façade plane is estimated from the 3D point cloud computed in the previous step (see Figure 2b). For estimation of the plane, we use a RANdom SAmple Consensus (RANSAC) estimator to obtain the best fit plane to the 3D points. The principle of the RANSAC estimator is to iteratively search for the best plane by fitting a random plane and computing distance from the given 3D points. Since the 3D point cloud is mainly generated from the TBF, the associated high inlier/outlier ratio produces rapid convergence to accurately find the plane’s location with the RANSAC estimator (the inliers are the points close to the TBF). Once the plane estimated, we need to recall the equation of a basic plane to find the 3D points on that plane. More precisely, any 3D points on a plane will satisfy the plane equation (e.g., ax + by + cz + 1 = 0). The plane estimated with the RANSAC estimator is denoted as π (1 × 4 vector) and the 3D points $X_{\pi }$, placed on this plane satisfy the equation:

(2) $$\pi X_{\pi }=0$$

Third, we compute a homography relationship between each of the images and the façade plane using projection matrices as shown in Figure 2c. The homography matrix is a 3 × 3 matrix providing a planar projection transformation between 2D images. This matrix thus provides a one-to-one mapping between the 2D points on the images and the 2D points on the estimated plane [24]. In the current coordinate systems, the points on the plane ($X_{\pi }$) are represented as all three values in each axis. To establish the homography relationship, one of the dimensions in $X_{\pi }$ should be reduced by transforming the coordinate system. A rotation matrix (R) transforms the current coordinate system to align its Z axis to the normal vector of the estimated plane π. Then, the plane in the new coordinate system ($\tilde{\pi }$), becomes parallel to the XY-plane, and a point on this plane $X_{\tilde{\pi }}$, has a constant value in the Z axis, which is $c_{\tilde{\pi }}$ in Equation (3). This relationship can be represented as:

(3) $$X_{\tilde{\pi }}=RX_{\pi }$$

where $X_{\tilde{\pi }}={\left[\begin{array}{cccc} a_{\tilde{\pi }} & b_{\tilde{\pi }} & c_{\tilde{\pi }} & 1 \end{array}\right]}^{T}$ and $\pi =\tilde{\pi }R^{-1}=[\begin{array}{cccc} {\tilde{\pi }}_{1} & {\tilde{\pi }}_{2} & {\tilde{\pi }}_{3} & {\tilde{\pi }}_{4} \end{array}]$. $X_{\tilde{\pi }}$ and $\tilde{\pi }$ in Equation (3) also satisfy the relationship in Equation (2), which becomes

(4) $${\tilde{\pi }}_{1}a_{\tilde{\pi }}+{\tilde{\pi }}_{2}b_{\tilde{\pi }}+{\tilde{\pi }}_{3}c_{\tilde{\pi }}+{\tilde{\pi }}_{4}=0$$

Any $X_{\tilde{\pi }}$ on the plane $\tilde{\pi }$ has a constant value of $c_{\tilde{\pi }}$ and will satisfy Equation (4). Notice that $\tilde{\pi }$ now can be expressed in 2D form, which means $c_{\tilde{\pi }}$ is not correlated with $a_{\tilde{\pi }}$ or $b_{\tilde{\pi }}$ and thus, $c_{\tilde{\pi }}$ yields:

(5) $$c_{\tilde{\pi }}=\;-{\tilde{\pi }}_{4}/{\tilde{\pi }}_{3}$$

Based on Equations (3) and (4), the homography matrix between $\tilde{\pi }$ and image *i* can be computed as:

(6) $$x_{i}=H_{i\tilde{\pi }}\times {\left[\begin{array}{ccc} a_{\tilde{\pi }} & b_{\tilde{\pi }} & 1 \end{array}\right]}^{T}$$

where $H_{i\tilde{\pi }}=\left[\begin{array}{ccc} {\tilde{p}}_{i}^{1} & {\tilde{p}}_{i}^{2} & c_{\tilde{\pi }}{\tilde{p}}_{i}^{3}+{\tilde{p}}_{i}^{4} \end{array}\right]$ and ${\tilde{P}}_{i}=P_{i}R^{-1}=\left[\begin{array}{cccc} {\tilde{p}}_{i}^{1} & {\tilde{p}}_{i}^{2} & {\tilde{p}}_{i}^{3} & {\tilde{p}}_{i}^{4} \end{array}\right]$, ${\tilde{p}}_{i}^{j}$ is the jth column of ${\tilde{P}}_{i}$. The homography matrix $H_{i\tilde{\pi }}$ maps the points on the plane $\tilde{\pi }$ to those on the image *i*.

Fourth, in Figure 2d, a set of the images is projected onto the facade plane using the homography matrices. In Section 2.1, although a large volume of images is collected from various viewpoints, rather than utilizing all the images it is best to only use a subset of suitable images for constructing the orthophoto. The backgrounds of the angled images do not have sufficient and regular resolution of the building regions and they may also include non-façade regions. Therefore, images that are relatively parallel to the TBF are selected to generate the orthophoto provided that they cover the entire area of the TBF. For the experimental validation in this study, we set a threshold for the angle between the normal vectors of the façade plane and the image planes at 20°. Note that although we use a set of mainly parallel images for orthophoto generation, the ROIs will be extracted from all images if they satisfy the constraints in Section 2.3.

Finally, the aligned projected images are stitched and blended to construct an orthophoto of the TBF, shown in Figure 2e. For their seamless composition, we implement gain compensation and multi-band blending developed by Allène, et al. [29]. Note that since the technique developed in this study assumes that scenes are placed on the same flat plane, scenes that are not captured from the plane of the TBF (e.g., ground, roof of the building or sky in this study) will be incorrectly wrapped (see Figure 2e) and not geometrically correlated to the images using Equation (6). In such cases, the engineers should simply ignore such unnecessary regions.

### 2.3. Region-of-Interest Localization

**Figure 3:**
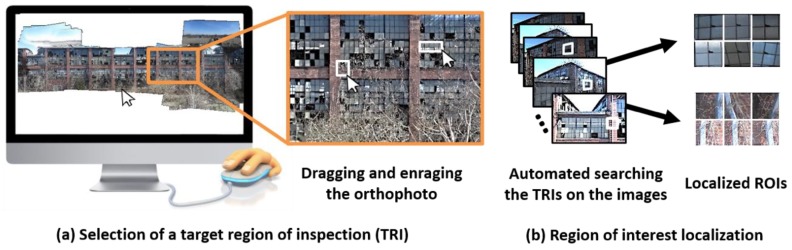
Region-of-interest localization procedure: (**a**) Selection of target regions of inspection (TRIs) and (**b**) Extraction of ROIs from raw collection of images.

The homography matrix between the orthophoto and each raw image can be computed using Equation (6). With this matrix, the ROIs, which are high-resolution image patches, corresponding to any region on the orthophoto can be extracted from the raw images. In this step, engineers are asked to define a TRI on the orthophoto, as illustrated in Figure 3. The user simply draws a polygon on the orthophoto that fully encompasses the TRI. Any region and shape of the polygon may be selected to define the TRI. For example, in our experimental validation we simply draw a rectangle by dragging the mouse to select damaged components on the TBF. A set of 2D points ($x_{i}$) in each image corresponding to the vertices of the selected polygon ($a_{\tilde{\pi }}$ and $b_{\tilde{\pi }}$) on the orthophoto can be computed from Equation (6). The portions of each image within those points becomes the ROI.

Here, not all images include a suitable ROI corresponding to the selected TRI because the ROI may not have a favorable condition that can be used for actual visual inspection. Thus, the following two conditions should be satisfied before the ROI is selected for use. First, the entire region of the selected TRI must be visible in each ROI. When an ROI is located at the boundary of an image it often does not contain a view of the entire TRI. Second, ROIs with very low quality (e.g., low resolution and motion blur) are not useful for visual inspection. Thus, the size of the ROIs should be larger than a pre-determined threshold that would produce the sufficient quality of the ROIs for visual inspection. For example, in the following experimental validation, the minimum ROI size is set to 200 × 200 pixels.

## 3. Experimental Validation

### 3.1. Description of the Test Building

**Figure 4:**
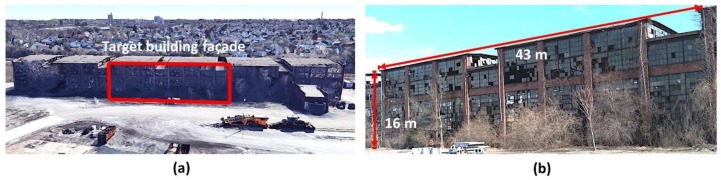
Experimental validation using a building having severe façade damage: (**a**) 3D view of the building (courtesy of Google 3D Maps) [31] and (**b**) Façade test region.

To demonstrate and validate the capability of the method developed herein, a building with severe façade damage (Figure 4) is utilized to reproduce the typical scenario of post-event building evaluation. This steel frame building is covered with window panes and masonry cladding on its TBF. The building is located in West Lafayette, IN, USA (GPS: 40.444922, −86.876579), and was originally built in 1925 as a railroad warehouse but has been abandoned since 1996 [30]. Thus, cracked or broken window panes, tiles and external drain pipes remain unfixed and untrimmed trees obstruct their views. The size of the façade of the test building is roughly 128 × 16 m^2^, but in this demonstration we only collect images from the center sections of the façade marked in Figure 4a. The size of the TBF is 43 × 16 m^2^ in Figure 4b. Window panes in the façade are opaque with an identical size of dimension 25.4 × 300 × 3 mm^3^.

### 3.2. Collection of the Images from the Test Building

**Figure 5:**
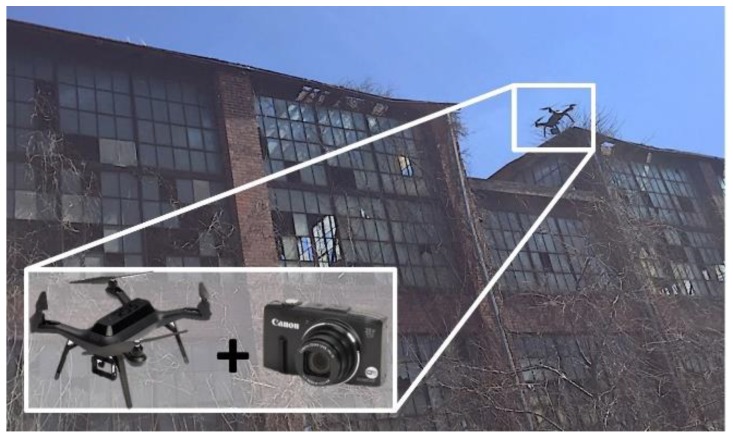
Image collection using an unmanned aerial vehicles (UAV) (3DR Solo Quadcopter) with a consumer-grade camera (Canon PowerShot SX280).

A consumer-grade UAV (3DR Solo Quadcopter, 3D Robotics, Berkeley, CA, USA) with a compact camera (Canon PowerShot SX280 HS, Canon Inc, Tokyo, Japan) is used for image acquisition in Figure 5. The cost of this equipment was around $1000 in 2015 but is becoming more affordable every year [32]. For high-quality image acquisition, we collect still images in continuous shot mode (1 frame/s) rather than taking a video [33]. Auto-focus or flash functions are not used. A total of 1254 images are collected from the TBF and the resolutions of all images are fixed to 4000 × 2664 pixels. Since this technique is developed for the purpose of rapid and automated image acquisition, the images are collected without advanced knowledge of damage locations or special control of camera angles in the middle of the flight. For successful implementation of the technique developed herein to other structures, users should design a proper image acquisition plan following the guidelines illustrated in Section 2.1.

**Figure 6:**
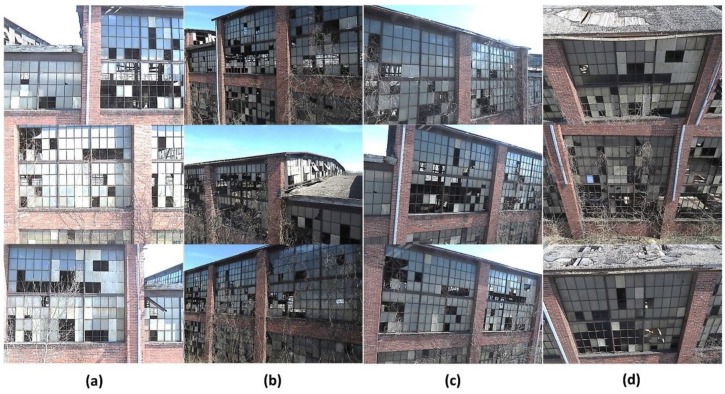
Sample images captured by UAVs from various angles: (**a**) Parallel, (**b**) left-angled, (**c**) right-angled, and (**d**) Downward.

In our experiment, suitable UAV flight paths are designed based on the guidelines established in Section 2.1. First, images are collected by flying the UAV along a grid pattern. During this process the UAV must first maintain a set altitude (vertical direction) and flies from one side to the other, and then repeats this process after changing its altitude. Image acquisition along this pre-designed flying path is assured to have sufficient and consistent overlap between the images in both the horizontal and vertical directions and to also capture the entire test façade. Second, the UAV must also maintain a constant and low flying speed to avoid abrupt transitions. Rapid transitions between the images will produce insufficient overlap between images and also makes the images blurry. The flying speed is determined based on the distance between the UAV and the test façade, the camera’s field of view (FoV), and the image collection rate. In this experiment, the distance is set to roughly 4–5 m to capture the detailed appearance of the test façade. The FoV and image capture rate of the camera are around 90° and one frame per second (1 frame/s), respectively. Accordingly, the flying speed is determined as 0.5–1 m/s to produce more than 60% overlap between images. Third, angled images must be collected from four different viewpoints. Sample angled images and parallel images are provided in Figure 6. In this experiment, we fly the UAV four times after changing the angles of the camera (parallel, right-and left-angled, and downward).

### 3.3. Results of Orthophoto Generation and Region-of-Interest Localization

In this study, VisualSfM is used to compute the projection matrix of each image [34]. VisualSfM is a freeware SfM software and provides a user-friendly graphic interface to monitor the intermediate steps of the SfM process, such as feature matching and camera pose estimation. VisualSfM highly improves the speed of the SfM computations by implementing the SiftGPU Library and parallel processing using Graphical Processor Units (GPUs) [35,36]. Once the projection matrices are computed from VisualSfM, the rest of the process including building plane detection and orthophoto generation are deployed in MATLAB [37]. In this experiment, it takes approximately four hours to process 1254 images to cover the 43 *×* 16 m^2^ TBF. A PC workstation having a Xeon E5-2620 CPU, Intel®, Holmdel, NJ, USA and NVidia Telsa k40c, Nvidia, Holmdel, NJ, USA with a 12 GB video memory GPU is used for this process. This period includes 53 min for image collection using the UAV, 3 h for computations associated with projection matrix estimation and point cloud generation, 0.2 h for façade plane estimation and image blending, and less than 1 min for the ROI localization once the engineers select the TRI. However, the time will vary depending on the size of the building façade and the number of images collected as well as the computation resources available and their specifications.

As a result, the orthophoto of the TBF in Figure 2e is successfully generated by automatically processing the raw aerial images. For the orthophoto, a total of 375 images (among 1254 images) are automatically selected as the set of images considered to be relatively parallel to the façade when the angle threshold is set to 20°.

**Figure 7:**
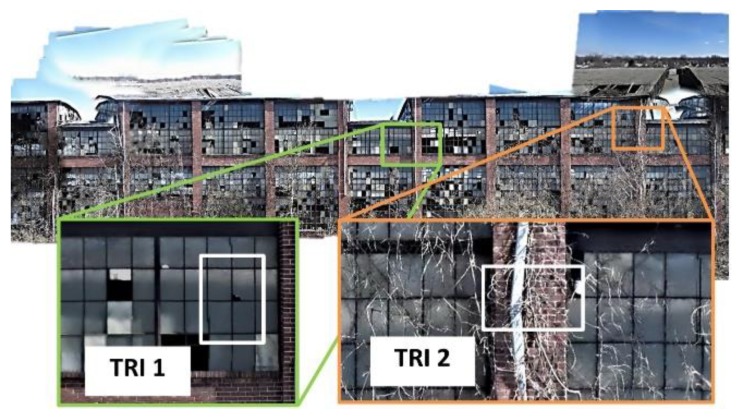
Selection of two sample TRIs (TRI 1 and TRI 2) on the orthophoto.

Figure 7 shows the constructed orthophoto of the TBF and a selection of sample TRIs. In this study, we choose two TRIs using rectangular boxes, denoted as TRI 1 and TRI 2. TRI 1 and TRI 2 include a broken window pane with a hairline crack and a cracked external drainage pipe, respectively. Although only two TRIs are chosen for this experimental validation, multiple regions with any sizes and locations may be selected with the technique developed. Note that the orthophoto is only used for assigning the TRIs. We recommend that further documentation and the actual inspection is conducted using the ROIs extracted from the original high-resolution images.

**Figure 8:**
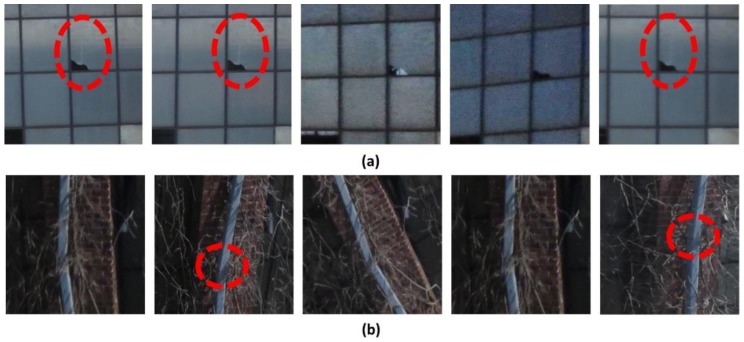
Localized ROIs corresponding to TRI 1 in (**a**) and TRI 2 in (**b**): The hairline vertical crack on a window pane in TRI 1 and damage on a drainage pipe in TRI 2 are only visible in specific ROIs and those damage locations are marked with a red dotted line.

Five samples of localized ROIs corresponding to each TRI are presented in Figure 8. The number of extracted ROIs corresponding to the two TRIs is 22 and 27 (from a total of 1254 images), respectively. All ROIs have different resolutions and aspect ratios depending on the depth between the images and TBF and viewing angles. However, in Figure 8, they are transformed into a square for this arrangement. These ROIs do satisfy the two conditions introduced in Section 2.3. Since the ROIs include details and multiple viewpoints of the TRIs, reliable vision-based visual inspection can be achieved. For instance, in Figure 8a, the white vertical crack propagated from the broken region is only visible in the specific ROIs captured from certain angles. Similarly, in Figure 8b, the break in the drainage pipe is only identified when the corresponding region is not impeded by the branch. The images that are captured with an angle are helpful in identifying such damage. These two examples clearly illustrate the need for collecting a sufficient number of images from different viewpoints and localizing the high-resolution ROIs from the original images for conducting robust visual evaluation.

## 4. Conclusions

In this study, we develop a vision-based approach for computer-aided rapid inspection for the TBFs. The technique developed here will automatically generate an orthophoto of the TBF using images collected from UAVs. First, UAVs collect a large volume of aerial images from the TBF by following the image collection guidelines developed in this study (see Section 2.1). Then, human inspectors select any region on the orthophoto where inspection is required and a set of ROIs corresponding to the region are localized from the high-resolution original images. Since the localized ROIs contain various viewpoints of the region, human inspectors can perform a complete and robust inspection. The feasibility of the method is demonstrated using an abandoned building having several damaged components on its TBF. ROIs corresponding to two TRIs having a damaged window pane and drainage pipe are extracted by processing a set of original images collected using a commercial UAV.

Some practical considerations are provided for successful implementation of the developed technique:

- Large geometric variations in the TBF (e.g., extrusions or intrusions), which are not placed within the same plane, will induce large distortions in the orthophoto. It is recommended in such a case that more images be captured parallel to the TBF and a smaller angle threshold be used for the orthophoto construction to reduce distortions due to a relief displacement coming from different elevations on the plane [37]. If the TBF does not lie within a single plane, engineers can generate multiple orthophotos and conduct visual inspection using each of the orthophotos. However, if the building façade is reasonably flat, a single orthophoto is sufficient to make the best use of the technique.
- As seen in Figure 8b, the presence of unwanted foreground objects (e.g., branch, tree, street light) may obstruct the view of the TRIs in the ROIs. In such a case, the only possible solution is to collect images from additional viewpoints. A similar issue occurs when the geometry of the structure is complex. Alternatively, one may further apply an image classification technique to filter out unnecessary ROIs and utilize only useful ROIs [21,23,38,39,40].
- In some cases, the existence of incorrect feature matches will introduce significant errors or even failures in the SfM process. The mis-associated features should be adaptively handled to enhance to the accuracy of the SfM outcomes. To address this issue, the authors have developed an adaptive resection-intersection bundle adjustment approach that refines the 3D points and camera poses separately [41].
